# Serum Uric Acid Level and Multiple Sclerosis: A Mendelian Randomization Study

**DOI:** 10.3389/fgene.2020.00254

**Published:** 2020-03-30

**Authors:** Peng-Peng Niu, Bo Song, Xue Wang, Yu-Ming Xu

**Affiliations:** Department of Neurology, The First Affiliated Hospital of Zhengzhou University, Zhengzhou, China

**Keywords:** multiple sclerosis, uric acid, mendelian randomization analysis, single-nucleotide polymorphism, causality

## Abstract

Previous observational studies have shown that the serum uric acid (UA) level is decreased in persons with multiple sclerosis (MS). We used the two-sample Mendelian randomization (MR) method to determine whether the serum UA level is causally associated with the risk of MS. We screened 26 single-nucleotide polymorphisms (SNPs) in association with serum UA level (*p* < 5 × 10^–8^) from a large genome-wide meta-analysis involving 110,347 individuals. The SNP outcome effects were obtained from two large international genetic studies of MS involving 38,589 individuals and 27,148 individuals. A total of 18 SNPs, including nine proxy SNPs, were included in the MR analysis. The estimate based on SNP rs12498742 that explained the largest proportion of variance showed that the odds ratio (OR) of UA (per mg/dl increase) for MS was 1.00 [95% confidence interval (CI) 0.90–1.11; *p* = 0.96]. The main MR analysis based on the random effects inverse variance weighted method showed that the pooled OR was 1.05 (95% CI 0.92–1.19; *p* = 0.50). Although there was no evidence of net horizontal pleiotropy in MR-Egger regression (*p* = 0.48), excessive heterogeneity was found via Cochran’s *Q* statistic (*p* = 9.6 × 10^–4^). The heterogeneity showed a substantial decrease after exclusion of two outlier SNPs (*p* = 0.17). The pooled ORs for the other MR methods ranged from 0.89 (95% CI 0.65–1.20; *p* = 0.45) to 1.05 (95% CI 0.96–1.14; *p* = 0.29). The results of sensitivity analyses and additional analyses all showed similar pooled estimates. MR analyses by using 81 MS -associated SNPs as instrumental variables showed that genetically predicted risk of MS was not significantly associated with serum UA level. The pooled OR was 1.00 (95% CI 0.99–1.02; *p* = 0.74) for the main MR analysis. This MR study does not support a causal effect of genetically determined serum UA level on the risk of MS, nor does it support a causal effect of genetically determined risk of MS on serum UA level.

## Introduction

The etiology of MS is varied and not fully understood (Dobson and Giovannoni, 2019). As a potent scavenger of peroxynitrite, previous studies suggested that UA may play an important role in the development of MS (Liu et al., 2012; Junqueira et al., 2017). An updated meta-analysis using data from case–control studies showed that the serum UA level was lower in persons with MS than in healthy controls (Wang et al., 2016). It has been suggested that the low serum UA level in persons with MS may represent the deficiency of protection against oxidative stress or the consumption of UA during anti-oxidative damage (Koch and De Keyser, 2006; Rentzos et al., 2006). However, the causal relationship between the serum UA level and the risk of MS remains unclear.

**FIGURE 1:**
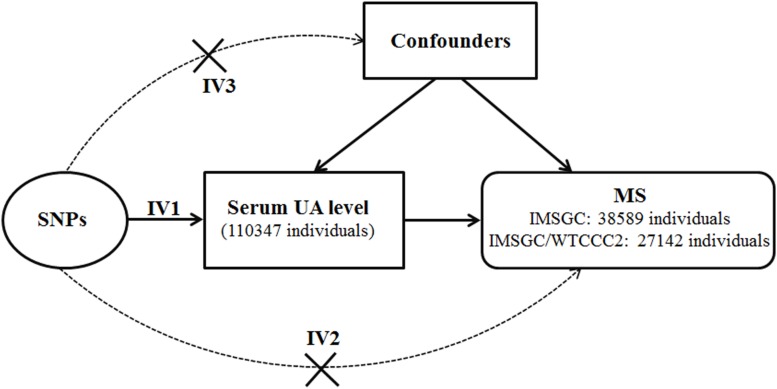
Diagram of Mendelian randomization analysis of serum uric acid level and multiple sclerosis. Abbreviations: MS = multiple sclerosis; SNP = single-nucleotide polymorphism; UA = uric acid. The three assumptions of the Mendelian randomization study are as follows: the SNPs associate with serum uric acid level (assumption IV1); the SNPs do not affect multiple sclerosis by other pathways (assumption IV2); and the SNPs are not associated with confounders (assumption IV3).

Because of the inherent bias, it is not possible to determine the direction of causality between the serum UA level and risk of MS from the above-mentioned case–control studies. The method of MR can be used to clarify the causality of exposure factors in disease etiology (Smith and Ebrahim, 2003; Mokry et al., 2015a; Harroud and Richards, 2018; Howell et al., 2018). In MR analysis, genetic variations such as SNPs will be used as surrogate measures of genetically determined lifetime exposure of the trait of interest. Because genetic variations are randomly allocated at meiosis, MR can mimic the design of randomized controlled trials and thus can solve the inherent bias of confounding and reverse causation in case–control studies (Smith and Ebrahim, 2003; Ong et al., 2018). There are three assumptions of the MR study (Figure 1). Hemani et al. (2018b) First, the genetic variations are directly associated with the exposure of the trait of interest. Second, the genetic variations do not affect the outcome by other pathways. Last, the genetic variations are not associated with confounders. The MR method has been used to determine the causal effects of many exposure factors on the risk of MS (Mokry et al., 2015b; Devorak et al., 2017; Harroud et al., 2019).

To determine whether a genetically associated serum UA level is causally associated with the risk of MS, we performed two-sample MR analyses using published data from large genetic studies including genome-wide meta-analyses.

## Materials and Methods

### Standard Protocol Approvals and Patient Consents

Approval and written consent for the present study were waived by the institutional review board of the First Affiliated Hospital of Zhengzhou University because the present MR analysis was based on summary data from previous genetic studies.

### Data Sources and Participants

For the exposure dataset, we screened 26 significant SNPs in association with serum UA level (*p* < 5 × 10^–8^) from a large meta-analysis of GWASs of serum UA level (Köttgen et al., 2013). The discovery analysis of this study comprises 110,347 individuals of European ancestry from 48 GWASs that have investigated variants associated with serum UA concentrations. In each of the 48 GWASs, genotype imputation was conducted using the HapMap 2 data as the reference. Across the 48 studies, mean serum UA concentrations ranged from 3.9 to 6.1 mg/dl (median of 5.2 mg/dl). Standard deviations of serum UA concentrations ranged from 0.92 to 1.68 mg/dl. The study reported that the proportion of variance in serum UA concentrations explained by these 26 SNPs was 7.0%. The proportion of variance in serum UA concentrations explained by the two leading SNPs (rs12498742 and rs2231142) was 3.4%.

The summary statistics of discovery data of these 26 target SNPs were used to perform the MR analysis. The summary statistics include the beta value and standard deviation of the effect of each SNP on serum UA concentrations. The information of effect allele, other allele, effect allele frequency, and sample size was also used to perform the MR analysis. SNPs were excluded from the MR analysis if their measured linkage disequilibrium had an *r* (Junqueira et al., 2017) greater than 0.01. We calculated the linkage disequilibrium using the CEU panel of the phase 3 data of the 1000 Genomes Project as the reference panel (The 1000 Genomes Project Consortium et al., 2015).

For the outcome dataset, we used the summary statistics of each target SNP for the risk of MS from the IMSGC ImmunoChip study and the IMSGC/WTCCC2 study (The International Multiple Sclerosis Genetics Consortium et al., 2011; International Multiple Sclerosis Genetics Consortium [IMSGC] et al., 2013). We used the summary statistics of the target SNPs regardless of whether the target SNPs were associated with the risk of MS or not. The IMSGC ImmunoChip study is a large genetic study regarding MS, which includes 14,498 MS cases and 24,091 healthy controls of European ancestry. If a UA-associated SNP was not available from the IMSGC ImmunoChip study, we searched the SNP from the IMSGC/WTCCC2 study, which includes 9,772 MS cases and 17,376 healthy controls of European ancestry. There were over 460,000 SNPs in the IMSGC/WTCCC2 study, which is substantially larger than those in the IMSGC ImmunoChip study (over 160,000 SNPs). If a UA-associated SNP was not available from both studies, a proxy variant was selected for the MR analysis. A genetic variant can be used as a proxy variant for the target variant if there is high linkage disequilibrium between them (*r*^2^ > 0.8). Proxy variants were searched from the IMSGC ImmunoChip study. If a proxy variant could not be found for a target variant from the IMSGC ImmunoChip study, we subsequently searched the IMSGC/WTCCC2 study. The summary statistics were chosen from discovery data of these two studies. Genotype imputation was not conducted for the discovery data in these two studies.

### Statistical Analyses

The estimate of UA on the risk of MS was evaluated using each SNP singly via the Wald ratio (Cheng et al., 2019). We used the random effects inverse variance weighted method to perform the two-sample MR analysis by pooling all of the estimates. Because horizontal pleiotropy (i.e. SNPs affecting the risk of MS through pathways other than the serum UA) may exist, a random effects model was chosen, as this model allows for each SNP to have different mean effects (Bowden et al., 2017). The estimates from each SNP and the pooled estimate were presented in a forest plot. The methods of the MR-Egger analysis, median-based estimator, and mode-based estimator were also used to perform the two-sample MR analysis. The MR-Egger analysis can produce an unbiased causal effect even if the assumption of no horizontal pleiotropy is violated for all SNPs (Bowden et al., 2015). The median-based method is a method that uses the median effect of all available SNPs, which could return an unbiased causal effect when at least half of the SNPs meet the assumption of no horizontal pleiotropy (Bowden et al., 2016). The mode-based method can return an unbiased causal effect if the SNPs within the largest cluster meet the assumption of no horizontal pleiotropy (Hartwig et al., 2017). SNPs that showed a similar causal effect will be grouped into the same cluster. ORs for MS were calculated per 1-mg/dl increase in serum UA level.

We performed leave-one-out sensitivity analysis to determine if the pooled estimate is being disproportionately influenced by each single SNP. We assessed heterogeneity among the estimates from each SNP via Cochran’s *Q* statistic. The presence of excessive heterogeneity suggests that some or all of the assumptions may be violated (Hemani et al., 2018b).

We performed all the statistical analyses in R (version 3.6.1) using the TwoSampleMR R package (Hemani et al., 2018b). The level of statistical significance was set at *p* < 0.05 (two-tailed).

We calculated statistical power using the method proposed by Brion et al. (2013) We had 80% power to detect an OR of 1.14 or 0.87 at an alpha rate of 5%.

### Data Availability

Publicly available datasets were analyzed in this study. Summary statistics can be found in NHGRIEBI GWAS Catalog.

## Results

### Variant Selection

Of the 26 target SNPs included, we identified six target SNPs from the IMSGC ImmunoChip study. Three target SNPs were further identified from the IMSGC/WTCCC2 study. Subsequently, we identified three proxy SNPs and six proxy SNPs from the IMSGC ImmunoChip and IMSGC/WTCCC2 studies, respectively. In total, we used 18 SNPs, including nine proxy SNPs, for the MR analysis. The two leading SNPs of rs12498742 and rs2231142 were included. We confirmed that there is no linkage disequilibrium (*r*^2^ < 0.01) between these SNPs. We found that none of the 18 UA-associated SNPs and the nine proxy SNPs were linkage disequilibrium (*r*^2^ ≤ 0.012) with any MS-associated SNPs that were reported in the two MS studies. We estimated that the total proportion of variance in serum UA concentrations explained by the 18 SNPs was ∼5.0%. We estimated that the *F* statistic was ∼322.6 using the proposed formula (Burgess et al., 2011) suggesting strong instruments for the present MR study (Burgess et al., 2011; Wang et al., 2019). Figure 1 shows the diagram of MR analysis of the serum UA level and MS. Table 1 shows the characteristics of SNPs included in the MR analysis.

**TABLE 1:** Characteristics of SNPs included in the Mendelian randomization analysis.

SNP	Chr	Closest gene	EA	OA	EAF^a^	SNP on serum UA (mg/dl)			SNP on MS			
						
						Beta	SE	*p*-value	OR (95% CI)	*p*-value	Study^b^	Proxy SNP
rs12498742	4	SLC2A9	A	G	0.77	0.373	0.006	0	1.00 (0.96–1.04)	0.961	IMSGC	rs7442295
rs2231142	4	ABCG2	T	G	0.11	0.217	0.009	1.0 × 10^–134^	0.99 (0.94–1.05)	0.795	IMSGC	–
rs1260326	2	GCKR	T	C	0.41	0.074	0.005	1.2 × 10^–44^	1.00 (0.96–1.03)	0.749	IMSGC	–
rs3741414	12	INHBC	T	C	0.24	–0.072	0.007	2.2 × 10^–25^	1.01 (0.97–1.05)	0.678	IMSGC	–
rs675209	6	RREB1	T	C	0.27	0.061	0.006	1.3 × 10^–23^	1.04 (1.00–1.08)	0.048	IMSGC	–
rs11264341	1	TRIM46	T	C	0.43	–0.050	0.006	6.2 × 10^–19^	1.03 (0.99–1.06)	0.109	IMSGC	–
rs653178	12	ATXN2	T	C	0.51	–0.035	0.005	7.2 × 10^–12^	0.95 (0.92–0.98)	0.002	IMSGC	–
rs1178977	7	BAZ1B	A	G	0.81	0.047	0.007	1.2 × 10^–12^	0.97 (0.93–1.01)	0.171	IMSGC	rs17145713
rs10480300	7	PRKAG2	T	C	0.28	0.035	0.006	4.1 × 10^–09^	1.02 (0.99–1.06)	0.267	IMSGC	rs10224002
rs1165151	6	SLC17A1	T	G	0.47	–0.091	0.005	7.0 × 10^–70^	0.94 (0.91–0.97)	3.8 × 10^–4^	WTCCC2	rs9393672
rs2078267	11	SLC22A11	T	C	0.51	–0.073	0.006	9.4 × 10^–38^	0.97 (0.94–1.00)	0.082	WTCCC2	–
rs7224610	17	HLF	A	C	0.58	–0.042	0.005	5.4 × 10^–17^	1.02 (0.99–1.06)	0.241	WTCCC2	–
rs6598541	15	IGF1R	A	G	0.36	0.043	0.006	4.8 × 10^–15^	0.99 (0.95–1.03)	0.606	WTCCC2	rs3743264
rs1394125	15	UBE2Q2	A	G	0.34	0.043	0.006	2.5 × 10^–13^	0.97 (0.93–1.01)	0.106	WTCCC2	–
rs7193778	16	NFAT5	T	C	0.86	–0.046	0.008	8.2 × 10^–10^	1.02 (0.97–1.07)	0.488	WTCCC2	rs33063
rs17050272	2	INHBB	A	G	0.43	0.035	0.006	1.6 × 10^–10^	1.01 (0.97–1.04)	0.718	WTCCC2	rs6706968
rs7188445	16	MAF	A	G	0.33	–0.032	0.005	1.6 × 10^–09^	0.97 (0.93–1.01)	0.160	WTCCC2	rs17767383
rs17786744	8	STC1	A	G	0.58	–0.029	0.005	1.4 × 10^–08^	1.00 (0.96–1.04)	0.900	WTCCC2	rs1705699

*CI = confidence interval; EA = effect allele; EAF = frequency of effect allele; MS = multiple sclerosis; OA = other allele; OR = odds ratio; SE = standard error; SNP = single-nucleotide polymorphism; UA = uric acid. ^a^Frequency of effect allele of uric acid dataset. ^b^IMSGC represents the IMSGC (International Multiple Sclerosis Genetics Consortium) ImmunoChip study; WTCCC2 represents the IMSGC/WTCCC2 (The International Multiple Sclerosis Genetics Consortium and the Wellcome Trust Case Control Consortium 2) study.*

### Estimates for Individual Variants

**FIGURE 2:**
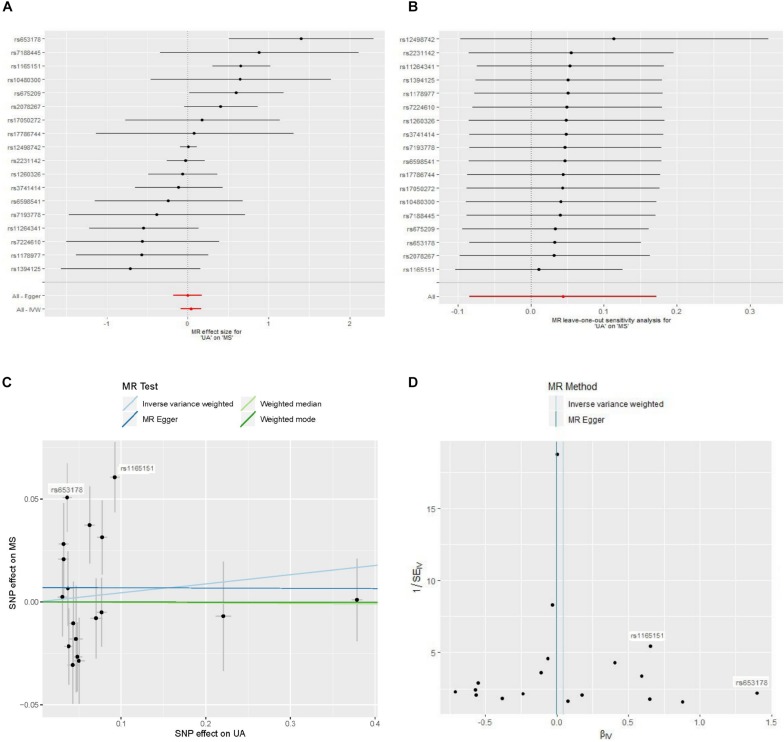
Forest plot, leave-one-out sensitivity analysis, scatter plot, and funnel plot of the effect of serum uric acid level on multiple sclerosis. Abbreviations: MR = Mendelian randomization; MS = multiple sclerosis; SNP = single-nucleotide polymorphism; UA = uric acid; IVW = inverse variance weighted. **(A)** Forest plot: The horizontal axis represents the estimate of serum UA level (log odds per 1-mg/dl increase) on MS. **(B)** Leave-one-out sensitivity analysis: Each black point represents the estimate of serum UA level (log odds per 1-mg/dl increase) on MS after the corresponding SNP was excluded. **(C)** Scatter plot: Each black point represents a SNP, plotted by the estimate of SNP on serum UA level (*x*-axis, 1-mg/dl units) and the estimate of SNP on the risk of MS (*y*-axis, log odds ratio) with standard error bars. The slopes of the lines correspond to causal estimates using each of the four different methods. **(D)** Funnel plot: Each black point represents a SNP, plotted by the estimate of serum UA level on the risk of MS on the horizontal axis and the inverse of the standard error of the estimate on the vertical axis. The vertical lines show the pooled estimates using two MR methods.

The estimates of serum UA level on risk of MS were significant for three SNPs (Figure 2A). The estimate based on SNP rs653178 showed that a genetically predicted 1-mg/dl increase in serum UA level was associated an increased risk of MS [OR 4.04; 95% CI 1.66–9.84; *p* = 2.1 × 10^–3^). The estimate based on SNP rs1165151 and rs675209 showed that the ORs were 1.92 (95% CI 1.34–2.76; *p* = 3.8 × 10^–4^) and 1.81 (95% CI 1.01–3.24; *p* = 0.046), respectively. The estimates based on the remaining SNPs showed that genetically predicted serum UA level was not associated with the risk of MS. The estimate based on SNP rs12498742 that explained the largest proportion of variance showed that the OR was 1.00 (95% CI 0.90–1.11; *p* = 0.96).

### MR Analysis

**FIGURE 3:**
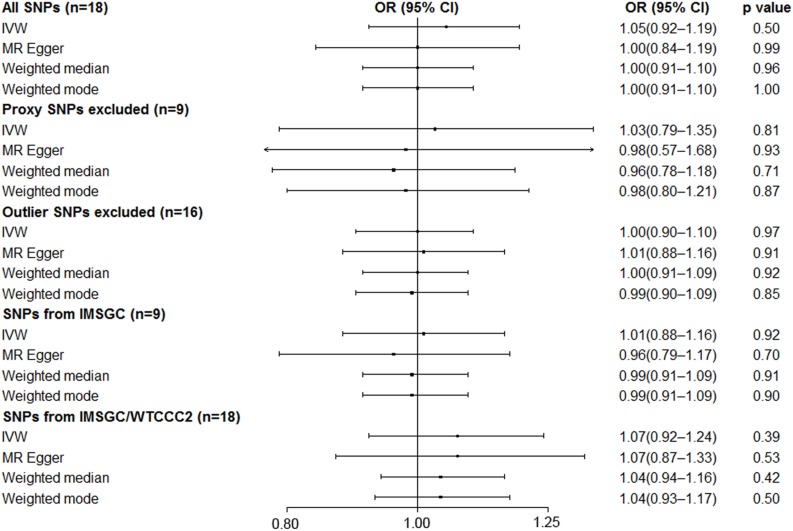
Mendelian randomization analyses of serum uric acid level on risk of multiple sclerosis. Abbreviations: CI = confidence interval; OR = odds ratio; IMSGC = International Multiple Sclerosis Genetics Consortium; IMSGC/WTCCC2 = The International Multiple Sclerosis Genetics Consortium and the Wellcome Trust Case Control Consortium 2; SNP = single-nucleotide polymorphism; IVW = inverse variance weighted. Cochran’s *Q* values for all SNPs, proxy SNPs excluded, outlier SNPs excluded, SNPs from IMSGC, and SNPs from IMSGC/WTCCC2 were 40.9 (*p* = 9.6 × 10^–4^), 23.2 (*p* = 3.2 × 10^–3^), 19.9 (*p* = 0.17), 19.5 (*p* = 0.01), and 49.3 (*p* = 5.5 × 10^–5^), respectively. The intercepts in the MR-Egger regression are 0.0070 (SE = 0.0097, *p* = 0.48), 0.0052 (SE = 0.0212, *p* = 0.81), −0.0017 (SE = 0.0077, *p* = 0.83), 0.0095 (SE = 0.0138, *p* = 0.51), −0.0006 (SE = 0.0113, *p* = 0.96).

MR analysis using a random effects inverse variance weighted method showed that a genetically predicted increase in the serum UA level (per 1-mg/dl increase) was not significantly associated with the risk of MS (OR 1.05; 95% CI 0.92–1.19; *p* = 0.50) (Figure 3). MR analyses based on the methods of MR-Egger analysis (OR 1.00; 95% CI 0.84–1.19; *p* = 0.99), median-based estimator (OR 1.00; 95% CI 0.91–1.10; *p* = 0.96), and mode-based estimator (OR 1.00; 95% CI 0.91–1.10; *p* = 0.99) showed similar results (Figure 3). The leave-one-out sensitivity analysis suggested that the MR analysis result was not driven dramatically by any single SNP (Figure 2B).

There was no evidence for a significant intercept (intercept = 0.0070; SE = 0.0097, *p* = 0.48) in the MR-Egger regression (Figure 2C), which suggests that there was no evidence of horizontal pleiotropy or that the horizontal pleiotropy is balanced.

Strong evidence of heterogeneity was found by Cochran’s *Q* statistic. The Cochran’s *Q* values were 40.9 (*p* = 9.6 × 10^–4^) for the random effects inverse variance weighted method. Asymmetry of the funnel plot also suggests evidence of heterogeneity (Figure 2D). After visual inspection of the forest plot (Figure 2A), scatter plot (Figure 2C), and the funnel plot (Figure 2D), we found two outlier SNPs (i.e. rs653178 and rs1165151) that may be the main sources of heterogeneity. The ORs remained unchanged after exclusion of the two outlier SNPs (Figure 3). Cochran’s *Q* values showed a substantial decrease of the heterogeneity after exclusion of the two outlier SNPs. Cochran’s *Q* values were 19.9 (*p* = 0.17) for the random effects inverse variance weighted method. There was no evidence of directional pleiotropy (intercept = −0.0017; SE = 0.0077, *p* = 0.83) in the MR-Egger regression after exclusion of the two outlier SNPs.

After excluding the nine proxy SNPs (i.e. nine SNPs left), the MR analyses showed similar results (Figure 3).

### Additional Analyses

**FIGURE 4:**
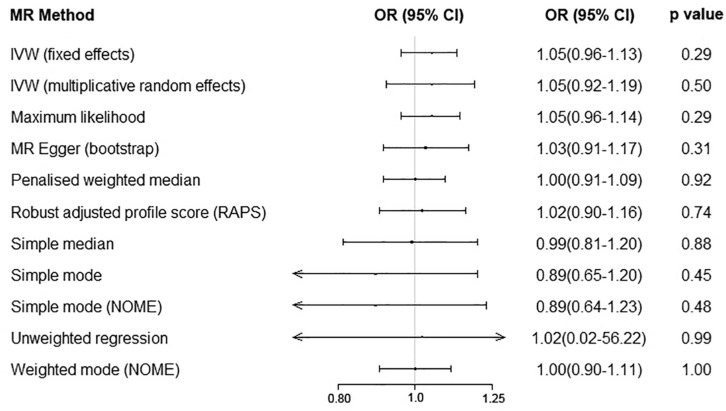
Serum uric acid level on risk of multiple sclerosis by using additional Mendelian randomization methods. Abbreviations: CI = confidence interval; OR = odds ratio; IVW = inverse variance weighted; MR = Mendelian randomization.

We performed the MR analyses by using the data from the IMSGC ImmunoChip study and the IMSGC/WTCCC2 study separately. All the results showed that a genetically predicted increase in the serum UA level (per 1 mg/dl) was not significantly associated with the risk of MS (Figure 3). We performed the MR analyses by using other MR methods. All the results showed no significant causal relationship (Figure 4).

**FIGURE 5:**
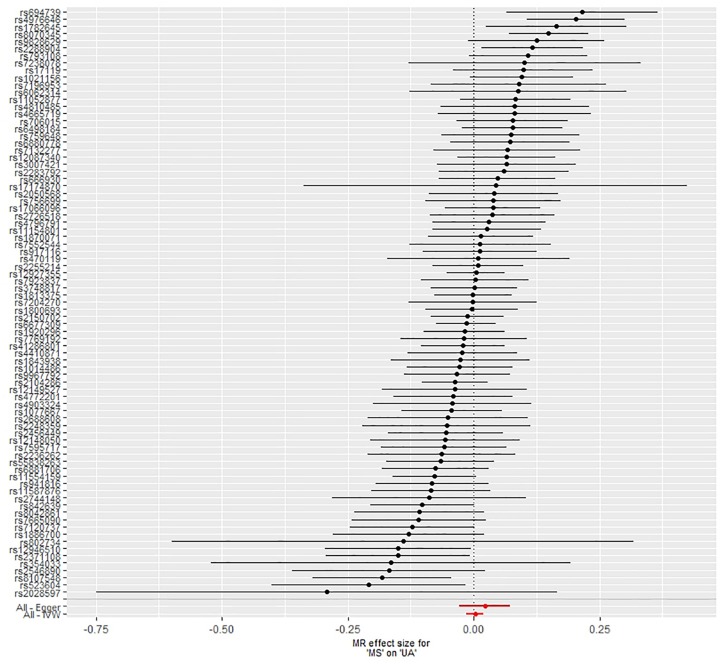
Forest plot of the estimate of risk of multiple sclerosis on serum uric acid level. Abbreviations: MR = Mendelian randomization; MS = multiple sclerosis; UA = uric acid; IVW = inverse variance weighted. The horizontal axis represents the estimate of multiple sclerosis risk on serum uric acid level.

**FIGURE 6:**
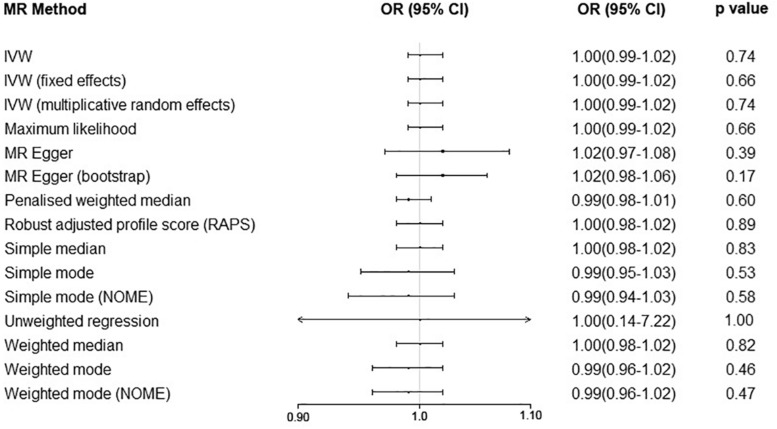
Mendelian randomization analyses of risk of multiple sclerosis on serum uric acid level. Abbreviations: CI = confidence interval; OR = odds ratio; IVW = inverse variance weighted; MR = Mendelian randomization.

Finally, we performed the MR analyses by using MS as the exposure and using serum UA as the outcome. We screened 110 significant SNPs in association with MS from the IMSGC ImmunoChip study. Seven SNPs were excluded due to linkage disequilibrium (*r*^2^ > 0.01) with other SNPs. Ninety of the 103 left SNPs were genotyped in the UA study. Nine SNPs were further excluded because of being palindromic with intermediate allele frequencies. In total, 81 SNPs including nine proxy SNPs were included in the MR analyses. MR analyses showed that genetically predicted risk of MS was not significantly associated with serum UA level (Figures 5, 6). There was no evidence of horizontal pleiotropy, or the horizontal pleiotropy is balanced (intercept = −0.0022; SE = 0.0027, *p* = 0.43). A strong evidence of heterogeneity was found by Cochran’s *Q* statistic (*p* = 2.2 × 10^–5^). We performed several sensitivity analyses. The results of leave-one-out analysis, MR analysis by excluding nine proxy SNPs, and MR analysis by excluding six outlier SNPs showed no significant causal relationship (data not shown).

## Discussion

In this MR study using the summary statistics of large genetic studies regarding serum UA levels and MS, we found no evidence to support a causal role of genetically predicted serum UA level for the risk of MS. The sensitivity analyses and additional analyses supported these findings. MR analyses also showed that genetically predicted risk of MS was not significantly associated with serum UA level.

Although there is increasing evidence of an association between decreased serum UA level and the risk of MS from case–control studies, the temporal order is unclear because of the inherent limitation of the case–control study and lack of prospective observational data. The present MR analysis suggests that even if there is an association between the serum UA level and MS, the observed decrease in the serum UA level is only an effect of MS disease attack, not its cause. In addition, the genetically predicted risk of MS was not significantly associated with serum UA level. Furthermore, although pilot clinical studies showed that treatment by increasing serum UA level have protective effects for persons with MS by reducing magnetic resonance imaging activity (Markowitz et al., 2009), preventing the progression of disease (Spitsin et al., 2001), and reducing relapse rates (Toncev, 2006), a subsequent multicenter double-blind placebo controlled trial with a 2-year study period failed to confirm these benefits (Gonsette et al., 2010). Although the sample size was small (*n* = 159), there was not even a slight trend of benefit on MS progression. This, in part, supports our finding that there is no causal relationship between genetically predicted serum UA level and genetically predicted risk of MS. The results of the present MR study support the idea that the low serum UA level in persons with MS may represent the consumption of UA during anti-oxidative damage after the disease attack, rather than a primary deficiency (Koch and De Keyser, 2006).

A recently published study reviewed relevant systematic reviews and MR studies that explored the causal associations of serum UA levels with multiple health outcomes (Li et al., 2017). Similar to our finding, the review showed that although serum UA levels were reported to be associated with most health outcomes in systematic reviews or meta-analyses of observational studies, most (84%) health outcomes investigated in MR studies were not statistically significant. This is because most MR studies could have been underpowered to detect modest effects. Our power calculation suggested that our MR analysis is sufficiently powered to assess an OR of 1.14 or 0.87. In addition, most of the point estimates from the pooled analyses in our study are very close or even equal to null, which suggests that there was probably no modest effect on risk of MS.

One important weakness of the MR study involves the second and third assumptions, which are difficult to evaluate (Hemani et al., 2018a). In our study, although no evidence of net horizontal pleiotropy was found using the MR-Egger regression (*p* = 0.90), evidence of heterogeneity suggested that one or more assumptions may be violated. After exclusion of the two outlier SNPs, the heterogeneity showed a substantial decrease and the ORs remained essentially unchanged. Furthermore, the results of different MR methods, sensitivity analyses, and additional analyses all showed similar results. Therefore, the pooled estimates in this study are probably unbiased or only slightly biased.

There are several limitations of this MR study which may bias the MR results. First, the first assumption of the MR study is that the instrument variable (i.e. UA-associated SNPs in this study) should be strongly associated with the exposure (i.e. serum UA level in this study). However, the proportion of variance in serum UA levels explained by the selected SNPs in our study was ∼5.0%, which was substantially lower than the heritability of 40–60%, suggested by a previous study (Krishnan et al., 2012). Weak instruments can result in misleading estimates of causal effects. Although the explained variance was relatively low, we estimated that the *F* statistic was ∼322.6, suggesting strong instruments for the present MR study (Burgess et al., 2011; Wang et al., 2019). Second, the IMSGC ImmunoChip study is a genetic study of MS risk that focused on immune-related variants outside the major histocompatibility complex, which may bias the MR results because non-immune-related variants were not investigated. A recently published genetic study involving more subjects found 200 autosomal susceptibility variants outside the major histocompatibility complex (International Multiple Sclerosis Genetics Consortium, 2019). Future updated MR analysis using the summary statistics of this larger genetic study is warranted to confirm the results of our MR study. The summary statistics of this study are not publicly available now. Third, the serum UA level that was measured at a specific time point may be affected by many temporary factors such as diet and UA-lowering therapies, which may not reflect the lifelong serum UA level determined by the encoding gene. However, these data were not available. Fourth, because the individual-level data were not available, we did not test the potential non-linear associations nor did we investigate the associations in different subgroups (e.g. males and females). Fifth, the results might also be influenced by survival bias by using case–control studies. Last, the included populations are all of European ancestry, which may restrict the generalization of the finding to other populations.

## Conclusion

In conclusion, by using the summary statistics from large genetic studies regarding serum UA level and MS, this MR study does not support a causal effect of genetically determined serum UA level on the risk of MS, nor does it support a causal effect of genetically determined risk of MS on serum UA level. The observed decreased serum UA level in MS persons in previous case–control studies may be an effect of the MS disease attack.

## Data Availability Statement

Publicly available datasets were analyzed in this study. Summary statistics can be found in NHGRIEBI GWAS Catalog.

## Ethics Statement

Ethical approval was not provided for this study on human participants because Approval and written consent for the present study were waived by the institutional review board of the First Affiliated Hospital of Zhengzhou University because the present MR analysis was based on summary data from previous studies. The ethics committee waived the requirement of written informed consent for participation.

## Author Contributions

P-PN and Y-MX conceptualized and designed the study and analyzed and interpreted the data. P-PN, BS, and XW collected and assembled the data. All authors wrote the manuscript and approved the final version of the manuscript.

## Conflict of Interest

The authors declare that the research was conducted in the absence of any commercial or financial relationships that could be construed as a potential conflict of interest.

## Abbreviations

CI: confidence interval
IMSGC: international multiple sclerosis genetics consortium
IMSGC/WTCCC2: the international multiple sclerosis genetics consortium and the wellcome trust case control consortium 2
MR: mendelian randomization
SNPs: single-nucleotide polymorphisms
GWASs: genome-wide association studies
MS: multiple sclerosis
OR: odds ratio
UA: uric acid.

## References

[B1] Bowden J. Davey Smith G. Burgess S. (2015). Mendelian randomization with invalid instruments: effect estimation and bias detection through egger regression. *Int. J. Epidemiol.* 44 512–525. 10.1093/ije/dyv080 26050253 PMC4469799

[B2] Bowden J. Davey Smith G. Haycock P. C. Burgess S. (2016). Consistent estimation in mendelian randomization with some invalid instruments using a weighted median estimator. *Genet. Epidemiol.* 40 304–314. 10.1002/gepi.21965 27061298 PMC4849733

[B3] Bowden J. Del Greco M. F. Minelli C. Davey Smith G. Sheehan N. Thompson J. (2017). A framework for the investigation of pleiotropy in two-sample summary data mendelian randomization. *Stat. Med.* 36 1783–1802. 10.1002/sim.7221 28114746 PMC5434863

[B4] Brion M. J. Shakhbazov K. Visscher P. M. (2013). Calculating statistical power in mendelian randomization studies. *Int. J. Epidemiol.* 42 1497–1501. 10.1093/ije/dyt179 24159078 PMC3807619

[B5] Buniello A. MacArthur J. A. L. Cerezo M. Harris L. W. Hayhurst J. Malangone C. (2019). The nhgri-ebi gwas catalog of published genome-wide association studies, targeted arrays and summary statistics. *Nucleic Acids Res.* 2019 D1005–D1012. 10.1093/nar/gky1120 30445434 PMC6323933

[B6] Burgess S. Thompson S. G. Collaboration C. C. G. (2011). Avoiding bias from weak instruments in mendelian randomization studies. *Int. J. Epidemiol.* 40 755–764. 10.1093/ije/dyr036 21414999

[B7] Cheng L. Zhuang H. Ju H. Yang S. Han J. Tan R. (2019). Exposing the causal effect of body mass index on the risk of type 2 diabetes mellitus: a mendelian randomization study. *Front. Genet.* 10:94. 10.3389/fgene.2019.00094 30891058 PMC6413727

[B8] Devorak J. Mokry L. E. Morris J. A. Forgetta V. Davey Smith G. Sawcer S. (2017). Large differences in adiponectin levels have no clear effect on multiple sclerosis risk: a mendelian randomization study. *Mult. Scler.* 23 1461–1468. 10.1177/1352458516681196 27903934

[B9] Giovannoni G. (2019). Multiple sclerosis - a review. *Eur. J. Neurol.* 26 27–40. 10.1111/ene.13819 30300457

[B10] Gonsette R. E. Sindic C. D’Hooghe M. B. De Deyn P. P. Medaer R. Michotte A. (2010). Boosting endogenous neuroprotection in multiple sclerosis: the association of inosine and interferon beta in relapsing- remitting multiple sclerosis (asiims) trial. *Mult. Scler.* 16 455–462. 10.1177/1352458509360547 20200198

[B11] Harroud A. Morris J. A. Forgetta V. Mitchell R. Smith G. D. Sawcer S. J. (2019). Effect of age at puberty on risk of multiple sclerosis: a mendelian randomization study. *Neurology* 92 e1803–e1810. 10.1212/WNL.0000000000007325 30894442 PMC6550505

[B12] Harroud A. Richards J. B. (2018). Mendelian randomization in multiple sclerosis: a causal role for vitamin d and obesity? *Mult. Scler.* 24 80–85. 10.1177/1352458517737373 29307294

[B13] Hartwig F. P. Davey Smith G. Bowden J. (2017). Robust inference in summary data mendelian randomization via the zero modal pleiotropy assumption. *Int. J. Epidemiol.* 46 1985–1998. 10.1093/ije/dyx102 29040600 PMC5837715

[B14] Hemani G. Bowden J. Davey Smith G. (2018a). Evaluating the potential role of pleiotropy in mendelian randomization studies. *Hum. Mol. Genet.* 27 R195–R208. 10.1093/hmg/ddy163 29771313 PMC6061876

[B15] Hemani G. Zheng J. Elsworth B. Wade K. H. Haberland V. Baird D. (2018b). The mr-base platform supports systematic causal inference across the human phenome. *eLife* 7:e34408. 10.7554/eLife.34408 29846171 PMC5976434

[B16] Howell A. E. Zheng J. Haycock P. C. McAleenan A. Relton C. Martin R. M. (2018). Use of mendelian randomization for identifying risk factors for brain tumors. *Front. Genet.* 9:525. 10.3389/fgene.2018.00525 30483309 PMC6240585

[B17] International Multiple Sclerosis Genetics Consortium (2019). Multiple sclerosis genomic map implicates peripheral immune cells and microglia in susceptibility. *Science* 365:eaav7188. 10.1126/science.aav7188 31604244 PMC7241648

[B18] International Multiple Sclerosis Genetics Consortium [IMSGC], Beecham A. H. Patsopoulos N. A. Xifara D. K. Davis M. F. Kemppinen A. (2013). Analysis of immune-related loci identifies 48 new susceptibility variants for multiple sclerosis. *Nat. Genet.* 45 1353–1360. 10.1038/ng.2770 24076602 PMC3832895

[B19] Junqueira S. C. Dos Santos Coelho I. Lieberknecht V. Cunha M. P. Calixto J. B. Rodrigues A. L. S. (2017). Inosine, an endogenous purine nucleoside, suppresses immune responses and protects mice from experimental autoimmune encephalomyelitis: a role for a2a adenosine receptor. *Mol. Neurobiol.* 54 3271–3285. 10.1007/s12035-016-9893-3 27130268

[B20] Koch M. De Keyser J. (2006). Uric acid in multiple sclerosis. *Neurol. Res.* 28 316–319. 10.1179/016164106x98215 16687059

[B21] Köttgen A. Albrecht E. Teumer A. Vitart V. Krumsiek J. Hundertmark C. (2013). Genome-wide association analyses identify 18 new loci associated with serum urate concentrations. *Nat. Genet.* 45 145–154. 10.1038/ng.2500 23263486 PMC3663712

[B22] Krishnan E. Lessov-Schlaggar C. N. Krasnow R. E. Swan G. E. (2012). Nature versus nurture in gout: a twin study. *Am. J. Med.* 125 499–504. 10.1016/j.amjmed.2011.11.010 22365026

[B23] Li X. Meng X. Timofeeva M. Tzoulaki I. Tsilidis K. K. Ioannidis J. P. (2017). Serum uric acid levels and multiple health outcomes: umbrella review of evidence from observational studies, randomised controlled trials, and mendelian randomisation studies. *BMJ* 357:j2376. 10.1136/bmj.j2376 28592419 PMC5461476

[B24] Liu B. Shen Y. Xiao K. Tang Y. Cen L. Wei J. (2012). Serum uric acid levels in patients with multiple sclerosis: a meta-analysis. *Neurol. Res.* 34 163–171. 10.1179/1743132811Y.0000000074 22333889

[B25] Markowitz C. E. Spitsin S. Zimmerman V. Jacobs D. Udupa J. K. Hooper D. C. (2009). The treatment of multiple sclerosis with inosine. *J. Altern. Complement. Med.* 15 619–625. 10.1089/acm.2008.0513 19425822 PMC3189001

[B26] Mokry L. E. Ahmad O. Forgetta V. Thanassoulis G. Richards J. B. (2015a). Mendelian randomisation applied to drug development in cardiovascular disease: a review. *J. Med. Genet.* 52 71–79. 10.1136/jmedgenet-2014-102438 25515070

[B27] Mokry L. E. Ross S. Ahmad O. S. Forgetta V. Smith G. D. Goltzman D. (2015b). Vitamin d and risk of multiple sclerosis: a mendelian randomization study. *PLoS Med.* 12:e1001866. 10.1371/journal.pmed.1001866 26305103 PMC4549308

[B28] Ong J. S. Gharahkhani P. An J. Law M. H. Whiteman D. C. Neale R. E. (2018). Vitamin d and overall cancer risk and cancer mortality: a mendelian randomization study. *Hum. Mol. Genet.* 27 4315–4322. 10.1093/hmg/ddy307 30508204

[B29] Rentzos M. Nikolaou C. Anagnostouli M. Rombos A. Tsakanikas K. Economou M. (2006). Serum uric acid and multiple sclerosis. *Clin. Neurol. Neurosurg.* 108 527–531. 16202511 10.1016/j.clineuro.2005.08.004

[B30] Smith G. D. Ebrahim S. (2003). ‘Mendelian randomization’: can genetic epidemiology contribute to understanding environmental determinants of disease? *Int. J. Epidemiol.* 32 1–22. 10.1093/ije/dyg070 12689998

[B31] Spitsin S. Hooper D. C. Leist T. Streletz L. J. Mikheeva T. Koprowskil H. (2001). Inactivation of peroxynitrite in multiple sclerosis patients after oral administration of inosine may suggest possible approaches to therapy of the disease. *Mult. Scler.* 7 313–319. 10.1177/135245850100700507 11724447

[B32] The 1000 Genomes Project Consortium, Auton A. Brooks L. D. Durbin R. M. Garrison E. P. Kang H. M. (2015). A global reference for human genetic variation. *Nature* 526 68–74. 10.1038/nature15393 26432245 PMC4750478

[B33] The International Multiple Sclerosis Genetics Consortium, the Wellcome Trust Case Control Consortium 2, Sawcer S. Hellenthal G. Pirinen M. Spencer C. C. (2011). Genetic risk and a primary role for cell-mediated immune mechanisms in multiple sclerosis. *Nature* 476 214–219. 10.1038/nature10251 21833088 PMC3182531

[B34] Toncev G. (2006). Therapeutic value of serum uric acid levels increasing in the treatment of multiple sclerosis. *Vojnosanit. Pregl.* 63 879–882. 10.2298/vsp0610879t 17121380

[B35] Wang L. Hu W. Wang J. Qian W. Xiao H. (2016). Low serum uric acid levels in patients with multiple sclerosis and neuromyelitis optica: an updated meta-analysis. *Mult. Scler. Relat. Disord.* 9 17–22. 10.1016/j.msard.2016.05.008 27645338

[B36] Wang X. Dai J. Y. Albanes D. Arndt V. Berndt S. I. Bézieau S. (2019). Mendelian randomization analysis of c-reactive protein on colorectal cancer risk. *Int. J. Epidemiol.* 48 767–780. 10.1093/ije/dyy244 30476131 PMC6659358

